# Play-mirth theory: a cognitive appraisal theory of humor

**DOI:** 10.3389/fpsyg.2024.1473742

**Published:** 2024-12-06

**Authors:** Leonidas Hatzithomas

**Affiliations:** Department of Business Administration, University of Macedonia, Thessaloniki, Greece

**Keywords:** humor theory, mirth, cognitive appraisals, reward, relief, playful turn, motive-consistency

## Abstract

This work aims to introduce a general theory of humor elicitation and appreciation, the play-mirth theory, which is based on the cognitive appraisal perspective. Two experiments test the theory’s central hypothesis: that is, to experience humor, one must interpret (a) a stimulus as a playful turn and (b) the turn as consistent with their motives. In the first experiment, 104 undergraduate students rated the appraisal determinants of successful and failed humor experiences that they recalled. In the second experiment, appraisals of playful turn (i.e., present or absent), situational state (i.e., motive-inconsistent/motive-consistent), and motivational state (punishment/reward) were manipulated. Overall, 150 undergraduate students were exposed to the manipulated stimuli and answered a structured questionnaire. The findings provide the first experimental evidence that two appraisals (i.e., playful turn and motive-consistency) do elicit humor. Play-mirth hypothesis sufficiently differentiates humorous from nonhumorous experiences as well as mirth from other positive emotions such as joy, and relief.

## 1 Introduction

Humor is a ubiquitous and important human activity (Martin and Ford, 2018; McGraw and Warren, 2010), a fundamental ingredient of social interaction (Wyer and Collins, 1992) that occurs across all cultures, social and age groups. People value humor as one of the most important factors in their lives (Oliveira et al., 2023), whereas they take into consideration “sense of humor” when selecting a mate (Morreall, 2020). Humor is, also, an integral element of work relationships and is positively associated with work-related performance, health, coping effectivity, job satisfaction (Scheel et al., 2016) and employee innovative behavior (Zhang and Su, 2020). Developmentally, laughter, the main behavioral response to humor, is one of the first social vocalizations, after crying, produced by infants (McGhee, 1979).

In the last 15 years, we have witnessed a proliferation of studies on humor (Martin and Ford, 2018). Numerous researchers in the fields of psychology (McGraw and Warren, 2010; Warren et al., 2021), sociology, anthropology, biology (Ramachandran, 1998), philosophy (Morreall, 2011), neuroscience (Amir et al., 2015), linguistics, literary, cultural, and business studies (Scheel et al., 2016; Hatzithomas et al., 2021; Zhang et al., 2022) have offered their insights into the role of humor in a wide range of human situations. However, a comprehensive theory explaining what makes something funny still remains the “holy grail” of humor research (Martin and Ford, 2018, p. 72).

Although the three classic theories of humor—incongruity, relief, and superiority—have introduced concepts widely accepted among humor researchers, these concepts are often too broad or vaguely defined and do not appear to constitute comprehensive theories of humor (Lintott, 2016; Martin and Ford, 2018; Morreall, 2020).^1^ The two contemporary theories with the most valid empirical foundation and the most significant implications, the reversal theory (Apter, 1982; Wyer and Collins, 1992) and the benign violation theory (McGraw and Warren, 2010), generated specific, and testable hypotheses. The two contemporary theories have significantly extended the prior works, systematized a set of underlying processes into a unifying structure, and offered persuasive explanations of the different humor phenomena. However, prior research has indicated that these two theories have limitations in explaining different forms of humor, and some of their terms and processes are rather unclear (Wyer and Collins, 1992; Hurley et al., 2013; Rozin et al., 2013; Raskin, 2017; Martin and Ford, 2018).

This article seeks to introduce a general theory of humor elicitation and appreciation, the play-mirth theory and invites the research community to engage in rigorous testing and validation. The theory proposes that humor occurs when and only when two conditions are satisfied: (a) a playful turn is perceived by an individual, and (b) the turn is considered consistent with the individual’s motivation. When these two appraisals occur simultaneously generate mirth, a short-lived, positive emotion and (usually) laughter. A playful turn is defined as a rapid and instantaneous cognitive shift from something viewed as (more) serious to something now seen as less serious (or more playful). Playful turn can be consistent with either an appetitive (i.e., reward-humor) or an aversive motive (i.e., relief-humor). The unexpectedness and relevance of the playful turn are humor enhancers, but they are neither sufficient nor necessary factors for humor elicitation and appreciation.

Two online experiments yielded the first evidence in favor of the play-mirth theory by testing its hypotheses. This research provides the following theoretical contributions: First, play-mirth theory appears to differentiate humorous from nonhumorous experiences sufficiently, exceeding the explanatory power of incongruity and benign violation theories. Second, it supports that mirth is a distinct positive short-lived emotion with its own appraisals; playful turn and motive-consistency. Third, it explains humor that is elicited both in playful and serious states suggesting two general types of humor, reward-humor and relief-humor. Play-mirth theory also leads to a non-tautological definition of humor as a rapid and instantaneous perception of life as less serious and more playful that elicits the positive short-lived emotion of mirth.

## 2 Literature review

### 2.1 Theories of humor

The three classic and the two contemporary theories of humor are briefly discussed below to underscore their main principles and limitations.

Superiority theory(ies) (LaFave, 1972; Gruner, 2017; Zillmann and Cantor, 2017), the earliest hypothesis about humor elicitation and appreciation, was based on the idea that humor is a form of aggression, and that mirth comes from a sense of triumph or superiority over other people. Several experimental studies have been conducted to test and support the superiority hypothesis, offering significant contributions to the humor literature (LaFave, 1972; Gruner, 2017; Zillmann and Cantor, 2017). The main criticism against this theory is that there is no evidence to support that all humor involves some form of aggression (Martin and Ford, 2018), and not every type of aggression or expression of superiority is inherently humorous (Morreall, 2020). Superiority theory fails to explain why witty remarks, innocent jokes, puns (Lintott, 2016), self-deprecating humor (Veatch, 1998), playful, absurd or nonsensical humor, slapstick, physical comedy (Morreall, 2011; Morreall, 2020), teasing and instances where people laugh with others are humorous. Consequently, Lintott (2016) argues that superiority theory is implausible as a comprehensive theory of humor.

Relief theory (ies) (Spencer, 1860) suggests that humor arises from the release or relief of excess nervous energy, psychological tension, and physiological arousal. Some contemporary theories are partly inspired by the relief theory’s perspective, such as Safron’s rapid anxiety reduction (RAR) theory (2019), which approaches humor as a negative emotion regulation strategy. The relief theory sheds light on the role of relief in humor appreciation but is not an all-encompassing theory of humor, since many laughter situations are not related to relief and do not involve the release of excess nervous energy (Martin and Ford, 2018). Today, nearly no scholars in psychology or philosophy describe laughter or humor as merely a mechanism for releasing superfluous nervous energy (Morreall, 2020). Even as a theory focused on anxiety reduction through tension release, the relief hypothesis falls short in explaining forms of humor that are valued for the cognitive pleasure derived from unexpected connections or absurd logic (Morreall, 2009).

The incongruity theory is the most widely accepted and extensively researched framework for humor creation and appreciation across psychology, social sciences, humanities, and business studies (Warren and McGraw, 2016; Attardo et al., 2024). According to the incongruity-resolution theory, humor is derived from the resolution of incongruities (Shultz, 1972; Suls, 1983). An incongruity is a situation or statement that contains a surprise or unexpected contradiction, deviation from the norm, or violation of established patterns or expectations. The cognitive process of recognizing and resolving an incongruity is critical to experiencing humor and is key to the incongruity resolution theory. Although incongruity resolution theory has provided substantial theoretical implications to the humor literature, there are some important limitations to its application and explanatory ability. For instance, prior research by Ruch and Hehl (1988) indicates that nonsense humor can be perceived as funny when sensation-seeking is involved, with no need for a resolution of the incongruity. Moreover, several studies have shown that unexpectedness is neither sufficient nor necessary for humor elicitation and appreciation (Topolinski, 2014; Warren and McGraw, 2016). Additionally, many pleasant and unpleasant incongruities are not funny (Martin, 2007), and the term *incongruity* is neither precisely nor consistently defined (Martin, 2007), which significantly reduces the explanatory power of the theory.

The McGraw and Warren’s (2010) benign violation theory posits that humor occurs when (1) a situation is appraised as a violation, (2) the situation is appraised as benign, and (3) both appraisals occur simultaneously. A violation is any stimulus that appears threatening, wrong, or negative and deviates from an individual’s perception of how things ought to be (Warren and McGraw, 2016). A benign appraisal occurs when a person perceives that there is nothing to worry about, meaning everything seems okay. Three conditions make a violation benign and thus humorous: (1) the presence of an alternative norm suggesting that the situation is acceptable, (2) weak commitment to the violated norm, and (3) psychological distance from the violation. This theory offers testable hypotheses and appears to explain several humor forms (Warren et al., 2021). However, benign violation theory cannot explain why some people find absurd humorous (Snow, 2014), while there is a variety of examples of benign masochism that can be viewed as benign violations but not funny (Rozin et al., 2013). Hurley et al. (2013) also question this theory’s rationale for explaining the humor generation process of puns as violations of grammar, spelling, or semantic rules, arguing that if any linguistic violation were inherently funny, then everyday language would be constantly amusing, given the frequent occurrence of such violations. In the same vein, Raskin (2017) argues that a benign violation is neither a necessary nor sufficient condition for humor, as numerous counterexamples demonstrate.

According to Apter’s (1982a) reversal theory of humor, a person must be in a playful motivational state and engaged in cognitive processes named as cognitive synergy and diminishment to experience humor. He theorizes that a cognitive synergy, that is, the attribution of incompatible qualities (e.g., real and apparent, sensible and stupid, honorable and dishonorable) to the very same identity (e.g., person/ object/ situation), is the basis of humor (Apter and Desselles, 2012). The synergy is perceived as humorous only when a change in the interpretation of the identity diminishes the identity’s value (e.g., an identity that was viewed as sensible is now seen as stupid). Diminishment can take place along many dimensions and at various levels of generality (e.g., person/person’s behavior/object/event/ situation/statement/conversation; Wyer and Collins, 1992) but can only be funny if a person is in a playful state of mind (Apter, 1982; Wyer and Collins, 1992; Apter and Desselles, 2012). This theory integrates cognitive and motivational processes but does not thoroughly address the emotional dynamics involved in humor elicitation and appreciation, such as the mirth that follows humor. Apter (1982a, p. 185) suggests that cognitive synergy may trigger a rapid motivational shift from a serious to a playful state of mind, implying that a playful motivational state is sometimes a result of humor rather than a prerequisite, which complicates the criteria of the theory. The reversal theory of humor also does not explain how humorous a situation would be when a joke teller uses cognitive synergy (e.g., a sensible/stupid synergy) that targets the joke hearer’s identity. Would the joke hearer perceive it as funny or as aggressive bullying? This scenario is common in playful states, such as during a game between children.

### 2.2 Play-mirth theory of humor

“*Humor is the instinct for taking pain playfully*”—Max Eastman (1922).

Play-mirth theory builds on prior frameworks, understands humor as play and puts the difference between serious and non-serious/playful in the center of its attention. As Max Eastman (1936, p. 15) argued “humor is play… therefore no definition of humor, no theory of wit, no explanation of comic laughter, will ever stand up, which is not based upon the distinction between playful and serious.” In the same vein, McGhee (1999) considers playfulness (and seriousness) as the foundation of the sense of humor. Seriousness and playfulness are often presented in the literature as two distinct individual differences (Proyer, 2017) and motivational states (Apter, 1982). The serious state is associated with essential/unavoidable goals imposed by society, family, and self-esteem, as well as a future orientation, whereas the playful state is associated with freely chosen, insignificant goals, and a present orientation (Apter, 1982). Milan Kundera (2020) writes that “the weight/lightness opposition is the most mysterious, most ambiguous of all” since it is difficult to categorize the two opposites as good or bad. He wonders whether we should live with “weight” and duty or avoid all external imperatives and live a life of lightness and freedom. There is probably no correct answer to this question because both weight (i.e., seriousness) and lightness (i.e., playfulness) can help humans survive, evolve, and thrive. Sometimes, however, the weight is unbearable and the switch to playfulness is more than necessary.

Seriousness is considered the default mode for humans, and humor offers a way to disengage from seriousness (Morreall, 2009, p. 255). Play-mirth theory suggests that any rapid and instantaneous transition from seriousness to playfulness (i.e., a playful turn) can create humor. This is the main difference between humor and the other forms of play. “Play is a free activity standing quite consciously outside ‘ordinary’ life as being ‘not serious,’ but at the same time absorbing the player intensely and utterly” (Huizinga, 1955, p. 13). Thus, while play is a non-serious mode of social interaction, humor is a play with the borders between seriousness and playfulness. This “walk on the cheerful edge” instantaneously expands the boundaries of personal freedom and is one of the primary goals of engaging in humor. Humor provides small doses of self-esteem and may have evolved to communicate that potential difficulties and external imperatives should not always be taken too seriously. Humor momentarily makes life seems more playful and easier than it really is.

#### 2.2.1 Playful turn

The central assumption of play-mirth theory is that one or more playful turns is the trigger of every comedic situation. The concept of playful turn has its origins in early philosophical thought about how individuals create humor. The term “eutrapelia” (Aristotle, Nicomachean Ethics, Book 2 87:2), in particular, was the first to be used to generally describe the phenomenon of playful turn. The Greek word “eutrapelia” combines the prefix “eu” meaning “easy” or “good,” and the word “trepo” meaning “turn.” That is, eutrapelia means “easy, good turn” and in ancient Greece referred to refined humor (Arneson, 2018). Eutrapelia was considered a virtue of happy and gracious flexibility in communication. Aquinas in his Summa Theologiae suggested that eutrapelia has social benefits and provides occasional rest. He extended the meaning of eutrapelia mentioning that “eutrapelos is a pleasant person with a happy cast of mind who gives his words and deeds a cheerful turn” (Morreall, 2009, p. 112).

**Figure 1:**
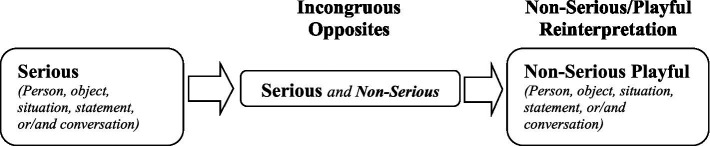
Playful turn as a cognitive process.

Play-mirth theory builds on these concepts (i.e., easy, good, and cheerful turn) and introduces the term “playful turn” to illustrate that humor is a form of play. Playful turn is defined as a rapid and instantaneous cognitive shift from something viewed as (more) serious to something now seen as less serious (or more playful). It is the reinterpretation of a (relatively) serious identity (i.e., person, object, situation, statement, or/and conversation) in a less serious (or more playful) way. In other words, humor involves bringing two cognitive opposites (i.e., a serious vs. a non-serious/playful) together so that they are both shown to be manifestations of the same identity. The juxtaposition of the two opposites is successive in a way that always leaves “a playful taste in the mouth” (i.e., a shift from a serious to a non-serious/playful interpretation; Figure 1). For the experience of humor, the shift from a serious to a non-serious/playful interpretation is required to be rapid in order to heighten the contrast between the two opposites and sharpen the transition. Humor has a dopamine-rewarding effect (Azim et al., 2005) and reward delays decrease dopamine responses to reward-predicting stimuli (Safron, 2019).

**Figure 2:**
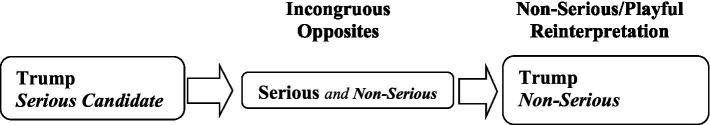
Example.

For instance, take into consideration the Barack Obama’s statement on Donald Trump’s potential candidacy for president: “[Donald Trump] can finally get back to focusing on the issues that matter, like, ‘Did we fake the moon landing?.” Obama’s statement appeared to be a compliment, but it was actually an irony of Trump’s seriousness as a candidate. There has been a rapid shift in the interpretation of Trump’s candidacy from serious to non-serious, that is, a playful turn (Figure 2).

**Figure 3:**
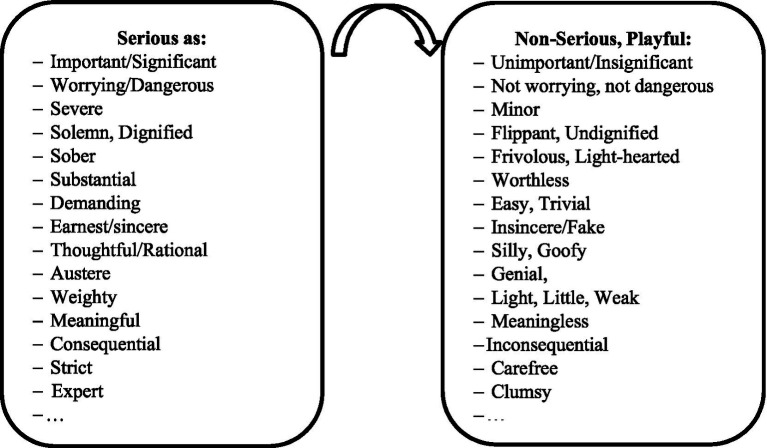
Playful turn.

The terms “serious” and “non-serious (playful)” must be understood using their different explanations, their synonyms, and the meaning of their synonyms (Figure 3). For instance, a turn in the meaning of an identity that shifts it from being viewed as thoughtful to being viewed as silly can cause humor. This (as every) playful turn is interpreted as a shift from something previously regarded as (more) serious to something now regarded as less serious (or more playful). There can also be multiple playful turns (e.g., severe/minor, sober/frivolous, important/unimportant) in an attempted joke. Only if the joke’s final total meaning expresses a shift from a (more) serious to a less serious interpretation is it humorous. Playful turns can occur both intentionally and unintentionally. For instance, a professional comedian tells jokes to make the audience laugh. On the contrary, both a professor who forgets to pull up his zippers, a dog that barks like a rooster and a vagina-like cave can make people laugh unintentionally. A playful turn can take more or less sophisticated forms, including metaphor, hyperbole, irony, sarcasm, overstatement, understatement, incongruity resolution, or simply the juxtaposition of two opposites. A playful turn can be people- (e.g., disparagement humor, sexist humor, and good-natured teasing) or non-people-oriented (e.g., food humor, scatological humor, witty wordplay, and malapropism), meaningful (e.g., the juxtaposition/synthesis of semantically relevant serious/playful opposites) or nonsense/absurd (e.g., the juxtaposition/synthesis of semantically irrelevant serious/playful components).

Humor play can involve either a novel/unexpected playful turn or a relevant “stock” playful turn. “Stock” playful turns are successful, playful turns that have been used in the recent past and may, therefore, continue to act as triggers of humor. Playful turn elicits a positive dopamine reward prediction error; that is, it offers a better reward than expected (see also Azim et al., 2005). It prepares the recipient for something serious, significant, and worrying that is ultimately non-serious and playful. The playful interpretation is considered as a reward for the recipient because either reduces the pressure and stress caused by serious, important, demanding, and threatening situations/identities or further increases positive thoughts and feelings. It instantly makes the situation appear more playful and easier for the recipient. It offers a greater reward than predicted and excites dopamine neurons. According to prior research (Schultz, 2015), when a stimulus A (e.g., serious identity) is paired with a reward (e.g., non-serious/playful identity) for the first time (e.g., unexpected/novel playful turn), prediction error signal and dopamine activity respond to the reward. During the next exposures to the same pair (i.e., relevant stock playful turn), the prediction error signal and dopamine activity respond to stimulus A (i.e., serious identity).

For example, let us suppose that we see Professor A and then notice that he forgot to pull up his zippers. The prediction error signal, dopamine activity, and perceived humor increase when we see his zipper down since we perceive the incongruous opposite between the serious and non-serious image of the professor (i.e., the playful turn). When we see Professor A again, the prediction error signal, dopamine activity, and perceived humor will all increase immediately, since we recall the playful turn. Also, in a conversation with students—who know Professor A’s funny event—about the seriousness of university lectures, it would be enough to say the name of Professor A to make them laugh. Of course, we eventually stop laughing at Professor A because humor wears out with repetition. Therefore, play-mirth theory suggests that both novelty/unexpectedness and relevance can increase humor appreciation.

Although, the terms playful turn and diminishment (as defined by Apter, 1982) share some similarities, have distinct differences. First, diminishment is a much broader term than playful turn. Many forms of diminishment cannot be classified as playful turns, such as a failure on an exam or a diagnosis with a serious illness. Even though these events are characterized as diminishments (see Apter and Desselles, 2012, p. 420), they cause a shift from a less serious interpretation to a more serious reinterpretation and hence they are not humorous. Second, diminishment can only be humorous if an observer is in a playful state of mind (Apter, 1982; Wyer and Collins, 1992; Apter and Desselles, 2012; Martin and Ford, 2018). On the other hand, a playful turn can occur if an observer is in either a playful or a serious state of mind. Even though a playful state of mind can function as a source of mirth, humor can flourish even under difficult circumstances. Austrian neurologist and psychiatrist Victor Frankl, reflecting on his experiences as an inmate in the Auschwitz concentration camp, argued that “Humor, more than anything else in the human makeup, affords an aloofness and an ability to rise above any situation, even if only for a few seconds” (Frankl, 2006, p. 63). In these cases, Apter (1982a) suggests that a joke can cause a rapid motivational switch from a serious to a playful state of mind. This, however, implies that a playful state of mind is a consequence of humor rather than a prerequisite.

#### 2.2.2 Motive-consistency

Playful turn is a cognitive process that replaces one attribute of an identity (i.e., seriousness) with another (i.e., reduced seriousness and/or increased playfulness). It is a necessary but not sufficient condition for experiencing humor. Playful turn needs to be combined with the appropriate motivation to produce a humorous response. Prior research on humor revealed not only the cognitive (Suls, 1983) but also the motivational mechanisms (Grüner, 1997; Zillmann and Cantor, 2017) that are involved in humor creation and appreciation. The primary motivations for humor have been proposed to be the release of tension/arousal (Freud, 1960), physiological arousal reduction (Berlyne, 1969), relief (Spencer, 1860), aggression (Grüner, 1997), self-esteem, superiority (LaFave, 1972; Zillmann and Cantor, 2017), inferiority reduction (Hatzithomas et al., 2021), and enjoyment (Apter, 1982). It is evident that most humor theories identify a single primary motive for the creation and appreciation of humor. This approach is very restrictive as it can only be applied within the bounds of the theories’ hypotheses. Superiority theories, for example, cannot account for puns, “innocent” and self-deprecating humor, relief theories cannot explain some incongruity resolution jokes, whereas both incongruity resolution theories and benign violation theory cannot easily account for absurd, non-sense humor (Martin and Ford, 2018).

Cognitive appraisal theory (a family of theories) as one of the most elaborate and influential theories of emotion (Moors, 2009; Pahng and Kang, 2023) can offer a complete framework for understanding the role of motivation on the generation of mirth (i.e., the emotion of humor). Cognitive appraisal theory proposes that unconscious, automatic cognitive appraisals are antecedents of emotion (Ellsworth and Scherer, 2003). A number of cognitive appraisals have been identified as important in the manifestation of different emotions, such as novelty/unexpectedness, situational state (motive-inconsistent/motive-consistent), motivational state (aversive/appetitive), probability, control potential, problem source, legitimacy, agency (Roseman et al., 1990), valence, certainty, and goal conduciveness (Ellsworth, 2013). This study employs Roseman’s Appraisal Theory of Emotions as its framework (Roseman et al., 1990). According to Roseman (1991), the interaction of situational and motivational states is the most important determinant of emotion intensity. The outcome of this interaction determines whether an emotion is positive or negative, as well as its intensity. A stimulus event that is consistent (inconsistent) with an individual’s motives generates a positive (negative) emotion. The motivational state, on the other hand, differentiates between contact-regulating (e.g., joy and sadness) and distance-regulating emotions (e.g., relief and distress).

**Figure 4:**
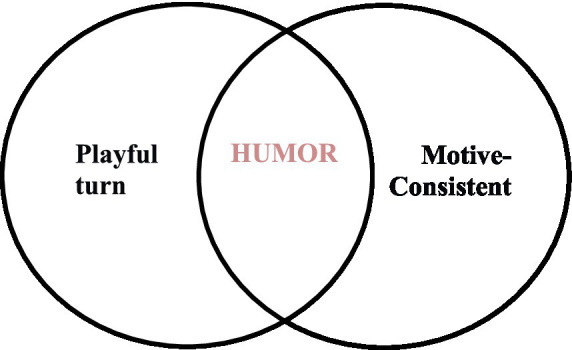
Play-Mirth theory.

Based on Roseman’s (1991) study, we can assume that mirth, as a positive emotion (Attardo, 2014), should be a response to a stimulus event appraised as consistent with an individual’s motives (Figure 4). Indeed, in cognitive appraisal literature, Scherer and Ceschi (1997) proposed that good humor is associated with a low goal/need obstructiveness, while Tong (2015) suggested that it is related to pleasantness (a dimension analogous to situational state according to Roseman et al., 1990). This perspective is also in accordance with both classic and contemporary motivational theories of humor. For instance, in superiority theories, the appreciation of humor is stronger when an individual has a positive disposition toward the victimizer and at the same time a negative disposition toward the victim of a joke (Zillmann and Cantor, 2017) and especially when the individual has a high superiority motivation (Hatzithomas et al., 2021). According to the relief theories (Spencer, 1860; Freud, 1960), humor facilitates psychological tension relief, a function that is consistent with an individual’s motives. Reversal theory also suggests that an individual appreciates humor and feels excited when their only goal in processing information is to understand and enjoy it (Wyer and Collins, 1992).

The play-mirth theory builds on Roseman’s Appraisal Theory of emotions and hypothesizes that humor occurs when a playful turn is perceived by an individual, and the playful turn is consistent with the individual’s motives. Contrary to prior research, it does not theorize that humor is only associated with the satisfaction of a specific motive. Motive-consistency refers to how much a playful turn is consistent with what an individual wants and how much it improves things for the individual (see also Roseman et al., 1996 for motive-consistency). Hence, a humorous playful turn can satisfy one or multiple motives at the same time, such as the need for enjoyment and excitement, the need for relief and the need for superiority.

Motive-consistency has significant differences with the concept of “benign” by McGraw and Warren’s (2010) benign violation theory. Motive-consistency occurs when a stimulus event (i.e., a playful turn) is consistent with our motives and not when it just makes us feel okay, as Warren and McGraw (2016) suggest when defining benign. It is not good in general, but good for us and our wants, and to be more precise, consistent with our wants. A joke that is “bad” for others and “good” for us may be humorous, whereas a joke that is “good” for others and “bad” for us probably not humorous. A joke that is inconsistent with our wants is definitely not humorous. For example, if we are trying to find an exit during an emergency evacuation, we are unlikely to laugh with a friend who is trying to tell a good “shaggy dog story.” If everything goes well, we may laugh at our friend’s attempt to tell us the joke, as our main goal will be to relax and recover from the shock.

Moreover, according to play-mirth theory, a stimulus event that is consistent with one motive but inconsistent with another is less humorous than a stimulus event that is consistent with one or more motives. A sexist playful turn, for instance, may be consistent with our need for enjoyment but inconsistent with our need to bond with a female friend and thus it is more likely to elicit less humor. In that sense, play-mirth theory predicts that benign violations may elicit mixed emotions rather than pure mirth.

#### 2.2.3 Appetitive or aversive motive

According to Roseman’s Appraisal Theory of emotions, both appetitive (i.e., pleasure-maximizing) and aversive (i.e., pain-minimizing) motives elicit positive emotions (Roseman et al., 1996). Appetitive motivational states, for example, are associated with the emotion of joy (when an individual attains a reward), whereas aversive motivational states with that of relief (when an individual avoids a punishment; Roseman, 1991). In the same vein, prior humor theories have approached mirth as a reward-seeking or stress-avoidance emotion. Incongruity theories (Suls, 1983), superiority theories (Zillmann and Cantor, 2017), and reversal theories (Apter, 1982; Wyer and Collins, 1992), for example, consider humor as a rewarding mechanism for generating pleasure, enjoyment, and self-esteem, whereas relief theories (Spencer, 1860; Freud, 1960), benign violation theory (McGraw and Warren, 2010), false alarm theory (Ramachandran, 1998), and RAR (Safron, 2019) view humor as a stress-avoidance mechanism for generating relief.

Prior studies have mostly failed to comprehend the two facets of humor and thus be too narrow to explain all instances of humor. Play-mirth theory asserts that the humor-generating appraisal process evaluates whether a person, object, situation, statement, or/and conversation is consistent or inconsistent with an individual’s motive(s); it is secondary whether the motive(s) is/are reward or relief-maximizing. The motivational state only distinguishes between reward and relief humor, two types of humor. In both cases, humor is considered a rewarding experience. Indeed, neuroscience studies have indicated that mirth activates classical reward/reward learning regions (Amir et al., 2015) and that the human brain treats relief in the same way that it does reward (Seymour et al., 2005).

Taking all together the present study formulates the following hypotheses:

*H1*: Mirth is experienced when both of the following conditions are satisfied simultaneously: (a) when an individual perceives a playful turn, and (b) the turn is considered consistent with the individual’s motive(s).^2^

*H2*: Unexpectedness has a positive effect on mirth.^3^

*H3*: Relevance has a positive effect on mirth.^4^

## 3 Study 1

### 3.1 Methodology

#### 3.1.1 Overview

We used the hypothetico-deductive method for theory testing (Eisend and Kuss, 2019). According to this approach, a general theory was developed, specific hypotheses were formulated, and based on the results, the hypotheses were evaluated to determine whether they were supported or rejected. The quantitative method, specifically experiments, is the most suitable approach for testing theories (Willer and Walker, 2007), as it allows for the manipulation of independent variables, objective measurements, statistical testing, causal inference, and comparisons across conditions, revealing nuanced differences in the effects of variables.

Study 1 tested hypotheses 1, 2, and 3. Following a standard and successful method for examining cognitive appraisals and emotions (Roseman et al., 1996; Rowe et al., 2019), we asked the participants to vividly recall humorous situations, describe them in their own words, and complete a questionnaire assessing their appraisals.

#### 3.1.2 Sample and procedure

Participants were 104 undergraduate students (57% females, *n* = 59) from a large Greek university. They were randomly divided into two treatment groups: We asked the first group’s participants to recall a successful joke someone told them when they were going through a difficult time (e.g., they were ill, had family or emotional problems, were preparing to take an exam), while in the second group’s participants a successful joke someone told them when they were having a good time (e.g., a time when they were with their friends and having fun). Then we asked them to also recall a failed joke they heard from someone under the same circumstances (Pundt et al., 2022). The order of successful and failed jokes was counterbalanced across participants. Thus, both successful and failed jokes were recalled approximately half the time as the first experience on a questionnaire and half the time as the second experience. To make the participants comprehend, we referred to a person’s failed attempts to tell a joke as a failed “joke.” However, this was not a joke nor humorous.

Gender was similarly distributed across the two groups: Females accounted for 57% (30 of 53) of those who recalled two jokes (a successful and a failed one) that someone told them when they were going through a difficult time and 57% (29 of 51) of those who recalled two jokes (a successful and a failed one) that someone told them when they were having a bad time. They ranged in age from 21 to 24, with a median age of 22. Participants in this study were recruited in exchange for extra class credit.

#### 3.1.3 Measures

The participants were given a questionnaire with two sections and the instructions listed below:

Treatment group 1 [2]: *“The first section of the questionnaire is about a successful joke that someone told you when you were going through a difficult time (e.g., you were ill, had family or emotional problems, were preparing to take an exam) [having a good time (e.g., a time when you were with your friends and having fun)]. It had to be a joke that arose from the conversation or situation at the time. Please describe in general terms the discussion/situation that preceded the joke. Please, also, describe the successful joke in detail.” “The second section of the questionnaire is about a failed joke that someone told you when you were going through a difficult time (e.g., you were ill, had family or emotional problems, were preparing to take an exam) [having a good time (e.g., a time when you were with your friends and having fun)]. It had to be a joke that arose from the conversation or situation at the time. Please describe in general terms the discussion/situation that preceded the failed joke. Please, also, describe the failed joke in detail.”*

After writing each joke, the successful or the failed one, the participants answered several questions about the appraisals that made them laugh at their recalled experiences. The stem “Successful/Failed joke was caused by” was given as the first part of each appraisal item to keep participants concentrated on the relevant appraisals. We adapted the scales developed by Roseman et al. (1996) to measure unexpectedness (e.g., Whatever person ‘A’ said was expected/unexpected, 3 items, Alpha = 0.77), motive consistency (e.g., Thinking that whatever person ‘A’ said was inconsistent/consistent with what I wanted, 5 items, Alpha = 0.97) and motivational state (e.g., From Wanting to get or keep something pleasurable to Wanting to get rid of or avoid something painful, 4 items, Alpha = 0.87). We also assessed relevance by using a 2-item scale (e.g., Whatever person ‘A’ said was/was not relevant to the prior conversation, Alpha = 0.96) and benign violation by using a 2-item scale (e.g., Seeing whatever person ‘A’ said as both wrong and not wrong, Alpha = 0.8). We defined the humorous responses as mirth to distinguish them from cognitive play (the combination of playful turn and motive-consistency). Mirth was measured through 3 items (i.e., It was funny, amusing, humorous). Four single-item measures were used to assess perceived joy, relief, laughter, and negative emotions.

We also used five questions to assess playful turns. The internal consistency of the questions “Thinking that what person ‘A’ said made the conversation/situation rapidly less/more serious for a brief moment”; “Thinking that what person ‘A’ said suddenly made something (e.g., a person, an idea, an institution, an animal, or object) less/more serious for a brief moment”; “Thinking that everything rapidly seemed less serious”; “Thinking that what person ‘A’ said made the conversation/situation suddenly less/more playful for a brief moment”; and “Thinking that what person ‘A’ said suddenly made something (e.g., a person, an idea, an institution, an animal, or object) less/more playful for a brief moment” was high (Cronbach’s alpha was 0.87). All items were measured on a 9-point Likert scale (see also Roseman et al., 1996). There were no missing values since all the questions were mandatory.

### 3.2 Results

A number of paired samples t-tests were conducted to analyze differences between successful and failed jokes (Table 1). Because 10 “assumptions” were tested in three different situations (i.e., in good and difficult times, and generally), a Bonferroni-adjusted significance level of 0.00167 was calculated to account for the increased possibility of type-I error. As expected, successful jokes elicited higher mirth [t(103) = 29.69, Cohen’s d = 2.91, *p* < 0.001], laughter [t(103) = 51.9, Cohen’s d = 5.09, *p* < 0.001], joy [t(103) = 36.52, Cohen’s d = 3.58, *p* < 0.001], relief [t(103) = 19.03, Cohen’s d = 1.87, *p* < 0.001], and lower negative emotions [t(103) = 20.32, Cohen’s d = −2.0, *p* < 0.001] than failed jokes. Also, the levels of all the appraisals were statistically higher in successful compared to failed jokes except for relevance [t(103) = 2.08, Cohen’s d = 0.2, *p* = 0.04]. Playful turn [t(103) = 16.21, Cohen’s d = 1.59, *p* < 0.001] and especially motive-consistency [t(103) = 35.44, Cohen’s d = 3.48, *p* < 0.001] had the most statistically significant differences between the two types of jokes. Unexpectedness [t(103) = 9.19, Cohen’s d = 0.9, *p* < 0.001] and benign violation [t(103) = 9.12, Cohen’s d = 0.89, *p* < 0.001] had also statistically significant differences between the two types of jokes. In successful jokes, the playful turn had the highest intensity (M = 7.72), whereas benign violation had the lowest (M = 4.92). In failed jokes, motive-consistency had the lowest levels (M = 2.03), while relevance had the highest (M = 5.06). Consistent with the Play-mirth theory, it appears that a playful turn can express the intensity of a humorous joke, and a motive-consistency can show when a joke is inappropriate.

**Table 1:** Paired samples *t*-tests.

	Total		Good times		Difficult times	
	Successful jokes	Failed Jokes	Successful jokes	Failed Jokes	Successful jokes	Failed Jokes
	M	M	M	M	M	M
Playful turn	7.72^**^	4.17	7.71^**^	4.39	7.73^**^	3.95
Motive-consistency	7.34^**^	2.03	7.04^**^	1.95	7.63^**^	2.1
Unexpectedness	7.22^**^	4.83	7.15^**^	4.79	7.28^**^	4.86
Relevance	5.91^*^	5.06	6.42	5.58	5.42	4.57
Benign violation	4.92^**^	2.54	5.21^**^	3.1	4.64^**^	2.39
Mirth	7.49^**^	2.09	7.54^**^	1.89	7.43^**^	2.29
Laughter	8.26^**^	1.69	8.26^**^	1.85	8.26^**^	1.53
Joy	7.27^**^	1.55	7.57^**^	1.66	6.96^**^	1.43
Relief	5.85^**^	1.41	5.66^**^	1.59	6.04^**^	1.22
Negative emotions	1.89^**^	7.02	1.66^**^	7.09	2.14^**^	6.94

*p < 0.05, **p < 0.001.

After dividing the data into binary variables (any score below 6 was transformed into 0, and the rest was 1, since 5 represented neutral) several chi-square tests were conducted to determine which appraisals could better differentiate between the successful and the failed jokes (Table 2). The participants were significantly more likely to perceive a playful turn (100%) in the successful jokes than in the failed ones (27.9%; χ^2^(1) = 117.29, Cramer’s V = 0.751, *p* < 0.001]. A playful turn was perceived in all successful jokes as an essential component of humor. Similarly, participants were more likely to perceive a motive-consistency in a successful joke (96.2%) than in a failed one [1.0%; χ^2^(1) = 188.64, Cramer’s V = 0.952, *p* < 0.001]. Interestingly, motive-consistency was perceived in nearly none of the failed jokes. The combination of these two appraisals, as predicted by the Play-mirth theory, can perfectly classify successful (96.2%) and failed jokes (1.0%; χ^2^(1) = 188.64, Cramer’s V = 0.952, *p* < 0.001], hence H1 is supported.

**Table 2:** Chi-squares.

	Total		Good times		Difficult times	
	Successful jokes	Failed jokes	Successful jokes	Failed jokes	Successful jokes	Failed jokes
	%	%	%	%	%	%
Play-Mirth Theory	96.2^**^	1.0	98.1^**^	0.0	94.1^**^	2.0
Playful turn	100.0^**^	27.9	100.0^**^	17.0	100.0^**^	39.2
Motive-consistency	96.2^**^	1.0	98.1^**^	0.0	94.1^**^	2.0
Unexpectedness	91.3^**^	43.3	92.5^**^	40.4	90.2^**^	51.0
Relevance	59.6	47.1	49.1	44.7	70.6^*^	47.1
Benign violation	43.3^**^	4.8	39.6^**^	6.4	47.1^**^	3.9

*p < 0.05, **p < 0.001.

Although both unexpectedness [91.3 vs. 43.3%; χ^2^(1) = 54.62, Cramer’s V = 0.512, *p* < 0.001] and benign violation [43.3 vs. 4.8%; χ^2^(1) = 42.13, Cramer’s V = 0.45, *p* < 0.001] were perceived mainly in successful rather than failed jokes, they were unable to perfectly distinguish between funny and nonhumorous stimuli. Hence, based on both T-test and Chi-square analyses, H2 is supported, as unexpectedness has a positive effect on mirth. Relevance was higher in the successful (59.6%) rather than in failed jokes (47.1%) but did not discriminate them since the difference was not statistically significant [χ^2^(1) = 3.27, Cramer’s V = 0.13, *p* = 0.071]. Participants were more likely to perceive relevance in a successful joke (70.6%) than in a failed (47.1%), only when they were going through a difficult time [χ^2^(1) = 5.83, Cramer’s V = 0.24, *p* = 0.016]. Therefore, based on both T-test and Chi-square analyses, H3 is partially supported, as relevance does not always positively affect mirth.

The findings also indicate that both appetitive (i.e., pleasure-maximizing) and aversive (i.e., pain-minimizing) motives elicit mirth. However, participants consider most of the jokes as reward humor (i.e., pleasure-maximizing; 72.1%) and less as relief humor (i.e., pain-minimizing; 27.9%). As predicted, reward humor was present especially during good times (good times = 65.3% vs. bad times = 34.7%), whereas relief humor was mainly during hard times [good times = 13.8% vs. bad times = 86.2%; χ^2^(1) = 22.23, Cramer’s V = 0.46, *p* < 0.001]. A correlation analysis of the variables studied is provided in Appendix 1.

### 3.3 Discussion

Study 1 gave support to the play-mirth theory and its main hypothesis that humor occurs when a playful turn is perceived by an individual, and the turn is considered consistent with the individual’s motive(s). Furthermore, it shows that unexpectedness and, to a lesser extent, relevance are humor enhancers (e.g., only during hard times), but they are neither sufficient nor necessary variables for eliciting and appreciating humor. Reward humor, also, prevails during good times, while relief humor during difficult times. Humor elicits solely positive emotions and not negative or mixed emotions. Interestingly, neither unexpected stimuli (i.e., incongruities) nor benign violations can perfectly classify successful and failed jokes, a result that shows the limited explanatory power of incongruity and benign-violation theories.

## 4 Study 2

Study 1 tested and supported the play-mirth theory. However, new research questions emerged that a second study should address. Motive-consistency appears to be the most important differentiator between successful and failed humor. Hence, the role of a playful turn on humor elicitation may be under question. Also, some authors have argued that studies on appraisal, by asking the participants to recall emotional experiences, may yield knowledge on the cognitive contents of emotional situations rather than their causes. Although we addressed this methodological problem by asking the participants to define what caused successful/failed humor, it would be better to conduct a second experiment that will manipulate appraisals and measure emotional responses.

### 4.1 Methodology

#### 4.1.1 Overview

Study 2 tested H1. As in seminal experiment of Roseman’s (1991), participants read brief stories about various protagonists’ experiences. In these stories, we manipulated the information relevant to the appraisals, and participants rated the intensity of the emotions (i.e., joy, relief, mirth, sorrow, and distress) that they believed the protagonists felt in response to the events.

#### 4.1.2 Experimental material and manipulations

Stories. Five-story scenarios with widely varying concrete content were created to assess generalisability. For example, one story was about a student attending an anatomy lesson at a university, and another was about an evening with friends at an Opera House.

Versions (appraisals manipulation): Each story was about a protagonist who was certain about the presence/absence (i.e., motive-consistency) of a rewarding/punishing state (i.e., motivational state). According to Roseman (1991), these two appraisals are associated with joy (presence of a rewarding state), relief (absence of a punishing state), sorrow (absence of a rewarding state) and distress (presence of a punishing state). One version of each story had a playful turn. Thus, five different versions of each story were constructed to manipulate appraisals. For instance:

Joy Version: Near John’s house, there is one of the most beautiful gardens in the village. In the center of the garden, five wonderful rose bushes surround the majestic statue of the village’s first mayor. John woke up early today to see the garden. John wants very much to see the rose bushes in bloom (rewarding). When John visits the garden, he notices that the roses are in full bloom (presence).

Sorrow Version: Near John’s house… in bloom (rewarding). When John goes to the garden, he notices that the rose bushes have been damaged by the wind (absence).

Relief Version: Near John’s house, there is one of the most beautiful gardens in the village. In the center of the garden, five wonderful rose bushes surround the majestic statue of the village’s first mayor. The wind is constantly destroying the rose bushes. John woke up early today to see the garden since it was very windy the night before. John does not want to see the rose bushes destroyed by the wind again (punishing). When John visits the garden, he notices that the roses are in full bloom (absence).

Distress Version: Near John’s house… the wind again (punishing). When John goes to the garden, he notices that the rose bushes have been damaged by the wind (presence).

Mirth Version: Near John’s house… the wind again (punishing). When John visits the garden, he sees that the roses are in full bloom (absence). He also notices on the head of the majestic statue of the village’s first mayor a white man’s underpants that probably left the laundry of the neighboring house when it was windy (playful turn).

#### 4.1.3 Design and sample

A 2 (motive-consistency: consistent/inconsistent) × 2 (motivational state: rewarding/punishing) factorial design was employed. Playful turn (presence/absence) was also used in three stories where a rewarding state was present and two stories where a punishing state was absent (i.e., whenever there was motive-consistency) since our main purpose was to differentiate mirth from other positive (i.e., joy and relief) and not negative emotions. Study 1 indicated that humor creates only positive emotions and not negative ones. When the 25 versions are grouped based on the emotions they produce according to the theory, a 5 (stories) × 5 (emotion manipulations) factorial design is obtained. As a result, our data can be analyzed in terms of the appraisal information contained within each version, or with respect to the emotion that each version (combination of appraisal information) elicits according to the theory.

Participants were 150 undergraduate students (53.3% females, *n* = 80) from a large Greek university. They recruited in exchange for extra class credit. Each participant read a set of five stories. Five “story sets” were created in such a way that: (1) each set contained one version of each of the five “story situations”; (2) only one version within a story set had an appraisal combination, and (3) only one version within a story set produced one of the five emotions. For each participant, the five stories of a set were randomly ordered.

#### 4.1.4 Measures

After carefully reading each story, the participants answered a few questions about the emotional responses of the protagonist based on their interpretation of the story. We told the participants that they could refer back to the story to assist them in answering the questions. As in the first study, we defined the humorous responses as mirth to distinguish them from cognitive play. The first question asked, “Which of the following emotions best describes how X (the protagonist) felt at the end of the story?” and provided the following choices: joy, relief, felt that it was humorous (mirth), sorrow, and distress. Next, there was another question asked, “How strongly did X (the protagonist) feel each of the following emotions at the end of the story?.” The participants had to define the emotional intensity (i.e., joy, relief, felt that it was humorous (mirth), sorrow, and distress) on an 11-point scale ranging from 0 (not at all) to 10 (very intensely; see also Roseman, 1991). There were no missing values, as all questions were mandatory.

### 4.2 Results

Table 3 summarizes the mean intensity ratings for each manipulated emotion. The intensity ratings along the main diagonal are higher than most of the ratings in the rest of the table, and hence, the data adheres to the hypothesized patterns well. We conducted a MANOVA on the mean intensity rating of emotions with manipulated emotions as the independent variable. The manipulated emotions variable had a value of Wilks’ Lambda of 0.085 and a *p*-value lower than 0.001 (*F*(4 2,459) = 135,83, *p* < 0.001; Wilks’ *Λ* = 0.085, partial eta square = 0.461), which means that the dispersion between groups is significant. The between-subjects effects and Bonferroni *post hoc* analyses indicated that the manipulated humor had statistically significantly higher mirth intensity than the other manipulated emotions (*F* = 607.2, *p* < 0.001; MH = 5.83, MJ = 0.97, MS = 1.23, MR = 1.33, MD = 1.76). As it was expected, manipulated humor also created joy (M = 6.57) and relief (5.83), since it shares some appraisals with them (i.e., motive-consistency and motivational state). Manipulated relief caused statistically significantly higher levels of relief than the other manipulated emotions. Thus, both manipulated humor and manipulated relief conformed totally to predictions. On the other hand, high levels of joy were generated by both the manipulated joy (M = 8.96) and the manipulated relief (M = 8.53). Similarly, the manipulated sorrow and the manipulated distress induced intense sorrow (MS = 8.25, MD = 8.07) and distress (MS = 7.07, MD = 7.49). These findings about joy, sorrow, relief, and distress are similar to those of Roseman’s (1991) study.

**Table 3:** Mean intensity ratings of theory-relevant emotions for all emotion manipulations.

	Emotion manipulated					*F* value, *p* value	Bonferroni *post hoc* tests
Emotion rated	Humor (H) *PL_+_, MC_+_, R_+_&P_−_*	Joy (J) *PL_−_, MC_+_, R_+_*	Relief (R) *PL_−_, MC_+_, P_−_*	Sorrow (S) *PL_−_, MC_−_, R_−_*	Distress (D) *PL_−_, MC_−_, P_+_*		
Mirth	**5.83** *SD = 3.42*	0.97 *SD = 1.57*	1.33 *SD = 2.1*	1.23 *SD = 2.2*	1.76 *SD = 2.66*	*F* = 607.2, *p* < 0.001	H > J, H > R, H > S, H > D
Joy	6.57 *SD = 3.25*	**8.96** *SD = 1.38*	8.53 *SD = 1.59*	0.66 *SD = 1.51*	0.46 *SD = 1.2*	*F* = 2,625.55, *p* < 0.001	J > H, J > S, J > D
Relief	5.83 *SD = 3.86*	7.32 *SD = 2.3*	**8.78** *SD = 1.6*	0.25 *SD = 0.68*	0.25 *SD = 0.9*	*F* = 2,405.81, *p* < 0.001	R > H, R > J, R > S, R > D
Sorrow	1.6 *SD = 2.46*	0.27 *SD = 0.97*	0.69 *SD = 1.66*	**8.25** *SD = 1.7*	8.07 *SD = 2.07*	*F* = 2,440.01, *p* < 0.001	S > H, S > J, S > R
Distress	1.7 *SD = 2.61*	0.25 *SD = 1.24*	0.7 *SD = 1.65*	7.07 *SD = 2.44*	**7.49** *SD = 2.44*	*F* = 1,881.54, *p* < 0.001	D > H, D > J, D > R

PL, playful turn present, MC, Motive-Consistency, R, Rewarding state, P, Punishing state, +, present, −, absent. The bold values in the table indicate the intensity ratings with the highest scores.

The participants were also asked to select which of the five emotions best describes how the protagonist felt at the end of the story. Table 4 presents the results of this analysis. The percentages along the main diagonal are higher than all the percentages in the rest of the table, and hence, the data adheres to the predicted patterns very well. We conducted a chi-square analysis to discern the relationship between manipulated emotions and participants’ emotion selections. The results of the Chi-square analysis indicate a very significant association between the manipulated emotions and the selections of the participants [χ^2^(16) = 1056.02, Cramer’s V = 0.593, *p* < 0.001]. We also performed adjusted standardized residuals to find which cells contributed to the chi-square test significance. If the standardized residual is higher than 2, a cell contributes significantly to the overall chi-square value. If it is less than 2, the cell contributes very little. Indeed, the participants recognized very well humor (Adj. std. resid. = 15.1), joy (Adj. std. resid. = 16.8) and relief (Adj. std. resid. = 15.3). They, however, appeared to face difficulties in differentiating between sorrow (Adj. std. resid._sorrow_ = 13.4, Adj. std. resid._distress_ = 9.4) and distress (Adj. std. resid._sorrow_ = 6.1, Adj. std. resid._distress_ = 8.9). Hence, based on both MANOVA and Chi-square analyses, H1 is supported. A correlation analysis of the variables examined is provided in Appendix 2.

**Table 4:** Matching theory-related emotions to the manipulated emotions.

	Emotion manipulated				
Emotion selections	Humor % (N) (*Adj. Residuals*) *PL_+_, MC_+_, R_+_&P_−_*	Joy % (N) (*Adj. Residuals*) *PL_−_, MC_+_, R_+_*	Relief % (N) (*Adj. Residuals*) *PL_−_, MC_+_, P_−_*	Sorrow % (N) (*Adj. Residuals*) *PL_−_, MC_−_, R_−_*	Distress % (N) (*Adj. Residuals*) *PL_−_, MC_−_, P_+_*
Mirth	**88.6** (62) *15.1*	0.0 (0) *−4.4*	1.4 (1) *−4.1*	1.4 (1) *−4.1*	8.6 (6) *−2.5*
Joy	18.5 (38) *−0.6*	**60.0** (123) *16.8*	20.5 (42) *0.2*	0.0 (0) *−8.4*	1.0 (2) *−8.0*
Relief	23.1 (39) *1.1*	14.8 (25) *−1.9*	**61.5** (104) *15.3*	0.0 (0) *−7.4*	0.6 (1) *−7.2*
Sorrow	2.1 (4) *−7.2*	0.0 (0) *−8.0*	1.0 (2) *−7.6*	**53.4** (102) *13.4*	43.5 (83) *9.4*
Distress	6.1 (7) *−4.1*	1.7 (2) *−5.3*	0.9 (1) *−5.6*	40.9 (47) *6.1*	**50.4** (58) *8.9*

PL, playful turn present, MC, Motive-Consistency, R, Rewarding state, P, Punishing state, +, present, −, absent. The bold values in the table represent the percentages with the highest scores.

### 4.3 Discussion

Study 2 demonstrated that mirth is a distinct emotion with its own appraisals; motive-consistency and playful turn. The playful turn differentiates mirth from the other positive emotions, such as joy and relief. Although the combination of playful turn and motive-consistency also generates joy and relief, it is the only combination that is considered humorous.

## 5 Conclusions and general discussion

The present paper aims to introduce a new theory for the humor elicitation and appreciation, the play-mirth theory. Two studies provided initial support for the theory’s explanatory power. Their findings suggest that appraisals of playful turn and situational state (motive-inconsistent/motive-consistent) differentiate between successful and failed humor. In study 1, 104 participants rated the appraisal determinants of their recalled successful/failed humor experiences. The study not only tested and offered initial evidence for the play-mirth theory’s predictions but also found that it outperforms the explanatory power of incongruity theory (i.e., unexpectedness) and benign violation theory (i.e., the opposition of good/right and bad/wrong). Warren and McGraw (2016) have observed that incongruity cannot distinguish between humorous and nonhumorous stimuli, whereas the benign violation hypothesis needs refinement since it sometimes fails, classifying nonhumorous stimuli as humorous. The play-mirth hypothesis builds on these important earlier theories and gives an explanation that can better differentiate between successful and failed jokes.

In Study 2, 150 participants read brief stories in which the appraisals of playful turn (i.e., present, or absent), situational state (i.e., motive-inconsistent/motive-consistent), and motivational state (punishment/reward) were manipulated. The findings show that mirth is a distinct emotion with its appraisals, playful turn, and motive-consistency. This contradicts previous research (Morreall, 2015) suggesting that humor amusement has not the basic features of standard emotions. Study 2 provides evidence that mirth is a short-lived positive emotion that shares certain appraisals with joy and relief.

The play-mirth theory integrates many of the ideas from the classic and contemporary theories and offers a new explanation that provides the following advantages:

Contrary to the classic theories of humor, and in line with the reversal theory and the benign violation theory it provides a basis for testable hypotheses. A testable theory is falsifiable by experiment and allows empirical validation and replication of the results, drives research for the revision and refinement of the framework and ultimately advances our understanding of the humor phenomenon (Martin and Ford, 2018).

Play-mirth theory integrates cognitive (i.e., playful turn), motivational (i.e., motivation consistency), and emotional processes (i.e., mirth) into a unified, coherent explanation of humor. Only the two contemporary theories of humor, reversal theory and benign violation theory, highlight the cognitive and motivational underpinnings of humor. They do not, however, go into great length about the effects of the humor process on perceived emotions. The play-mirth theory builds on the cognitive appraisal framework and describes the appraisals that cause the emotion of mirth (i.e., the emotion of humor). In this manner, the play-mirth theory can differentiate mirth from other positive emotions such as joy, and relief. It theorizes mirth as a distinct positive emotion and hence contradicts benign violation theory, which proposes that humor induces mixed emotions (McGraw and Warren, 2010). Study 1 supports that only failed humor attempts may induce negative emotions. The play-mirth theory also reveals the interplay between cognition and emotion in the elicitation and appreciation of humor, thereby contributing to the field of cognitive psychology, which seeks to understand this complex dynamic.

Past theories propose that humor occurs either during a playful state, such as reversal theories (Apter, 1982; Wyer and Collins, 1992), or during a negative situation, such as relief theories (Spencer, 1860; Freud, 1960), benign violation theory (McGraw and Warren, 2010; i.e., when an individual experiences a threat), false alarm theory (Ramachandran, 1998), and RAR (Safron, 2019). Play-mirth theory can explain humor that is generated not only in a playful, positive state but also in a serious, negative state of mind. During a positive situation, reward-humor seeks to prolong the pleasant moments, while during a negative situation, relief-humor tries to lighten difficult times.

The new humor theory also accounts for the critical role of timing in humor appreciation. For a playful turn to be perceived as humorous, it must align with an individual’s current desires or motives. Thus, the ideal timing for a joke-teller is when their interlocutor is inclined to satisfy specific motives. At this moment, the joke-teller delivers a playful turn that resonates with the joke-hearer’s motives, enhancing the likelihood of a mirthful response. By understanding and responding to the listener’s situational state, humor can be more effectively crafted to foster shared enjoyment, strengthen interpersonal bonds, and build social connections. In doing so, the theory also sheds light on the social aspects of humor.

The play-mirth theory also leads to a non-tautological definition of humor: Humor is the rapid and instantaneous perception of a serious person/object/event/ situation/statement/conversation as less serious (or/and more playful) that elicits the positive short-lived emotion of mirth. In general, humor is the rapid and instantaneous perception of life as less serious (or/and more playful).

## 6 Practical implications

Play-mirth theory has several practical implications at both the individual and organizational levels. At an individual level, people who struggle to understand or create humor may experience challenges in their social interactions, particularly with non-intimates (Bell, 2015; Martin and Ford, 2018). Play-mirth theory can explain why it is easier to create humor with friends, relatives, and romantic partners than with strangers (Bell, 2015), as we are generally more familiar with the motives of those close to us. Miczo and Averbeck (2020) also found that in romantic relationships, a partner’s use of negative relational humor predicts decreased relationship satisfaction for the other partner due to increased relational uncertainty, whereas positive relational humor is linked to greater relationship satisfaction through reduced relational uncertainty. Overall, inappropriate humor can create distance between romantic partners, undermining the mechanisms that nurture and sustain a relationship. For instance, failed interpersonal affect regulation resulting from inappropriate humor reduces a person’s confidence and motivation to engage in future attempts to influence others’ emotions (Williams and Emich, 2014). Play-mirth theory can illuminate the humor process as a play with the boundaries between seriousness and playfulness among people who share mutual understanding. In this manner, it can help reduce humor failures that impact social and romantic relationships both in the short term and over the long run.

Moreover, this theory can help professional coaches effectively guide their coachees by providing a deep yet accessible understanding of humor elicitation and appreciation. The concept of playful turns (a rapid change from something perceived as serious to something seen as less serious) is conceptually simpler to understand and create compared to benign violations (the perception of something as both wrong and not wrong) and cognitive synergies (which involve an interpretation that diminishes the value of an identity). Complex descriptions of the humor creation process can lead to highly creative attempts that often fail to generate mirth (Bell, 2015). Additionally, the concept of motive consistency directs coaches to help coachees build empathy to recognize when a playful turn aligns with their interlocutors’ desires. Improving the coaching process can have a significant implication, as humor can deepen the working alliance, foster adaptive coping mechanisms in clients, and enhance cognitive and behavioral processes (Vendl et al., 2024).

In general, a theory that explains how to create successful humor has important implications for workplace relationships and leadership roles. Previous studies have indicated that unsuccessful humor attempts can harm workplace status by negatively impacting perceptions of the joke-teller’s competence (Bitterly et al., 2017). Failed humor also shows a negative correlation with leader-member exchange—the unique, two-way relationship that develops between a leader and each team member (Pundt et al., 2022) while also decreasing follower liking toward the leader and follower advice-seeking (Ji et al., 2023). Humor shapes interpersonal perceptions and hierarchies within groups and as such is considered an important managerial skill (Bitterly et al., 2017). Play-mirth theory can help managers, leaders, and teams create appropriate humor to build mutually beneficial relationships in the workplace.

This theory has practical implications for advertising practitioners as well. Advertisers often use rhetorical figures—schemes and tropes—to create humor and stimulate cognitive pleasure (Margariti et al., 2022). However, it is known that not all rhetorical figures are inherently humorous (Dynel, 2009, 2014). Play-mirth theory can assist advertising professionals in distinguishing between humorous and non-humorous metaphors, ironies, hyperboles, and antitheses by applying the concept of playful turn. It also aligns with advertising practices to adapt messages to the desires and wants of target groups by emphasizing the need for motive-consistency in humor. Advertising practitioners should have a thorough understanding of their target audience to develop messages that generate humorous reactions effectively.

## 7 Limitations and future research directions

The concept of playful turn can direct researchers’ attention to the underlying mechanisms of humor rather than just its different forms. The emphasis of prior studies on different types of incongruity resolution (Suls, 1983; Wyer and Collins, 1992) and simultaneity (Warren et al., 2021) may be associated with the efficacy of these techniques to create a rapid shift from something more serious to something less serious (or more playful) generating successful jokes. However, spontaneous conversational humor can take various forms, such as “stock conversational witticisms,” namely humorous sayings used often in everyday conversations, that owe their effectiveness mainly to exaggerated facial expressions or bodily gestures and their relevance rather than their unexpected content (Martin and Ford, 2018). Future research could measure the velocity at which different types of humor create playful turns and its effect on mirth intensity, assessing in parallel the role of contextual and nonverbal factors and the influence of relevance in this relationship.

The concept of motive-consistency can help researchers to understand why humorous attempts often operate as a double-edged sword and explain part of the “bad” reputation of humor. According to play-mirth theory, humor is not associated with the satisfaction of a specific motive. Instead, a playful turn should be consistent with what an individual wants in order to be humorous. Hence, if one wishes to create humor in social interactions, one should be aware of what the other people want at that time (i.e., their motivation). Understanding other people’s sensitivities, desires, and preferences might assist one in determining whether a joke is appropriate or seen as improper and offensive. It is not all about the technique of humor, rather it is also about empathy and interpreting/understanding social situations and dynamics. This idea can notably influence research on disparagement humor (Baltiansky et al., 2021), where understanding people’s motives may be the difference between the success or failure of a joke.

Play-mirth theory can serve as inspiration for a more comprehensive leader humor expression construct, encompassing two distinct humor expressions: reward and relief humor. Such a framework could significantly contribute to the evolution of the organizational psychology research field, aiding leaders in effectively using humor to foster team cohesion, manage stress, and enhance internal communication. It can enable researchers to use clear, concise, specific, and expressive language to describe humor use in construct items, avoiding direct terms such as “humor,” “laughter,” “joke,” “amusement” or “mirth.” This approach will help reduce potential response bias induced by the scale. According to Kong et al. (2019), existing measures of leader humor styles, and especially those related to leader humor expression, often cover a narrow range of humor and present several conceptual problems.

Moreover, organizational psychology research often focuses on the differential effects of positive and negative humor on employees’ satisfaction and organizations’ effectiveness (Pham and Bartels, 2021). The constructs measuring positive and negative humor tend to emphasize the outcomes of humor use (e.g., “The humor used by my coworkers makes someone in the group feel bad”) rather than focusing on the underlying mechanisms of humor (e.g., an alternative could be: “The humor used by my coworkers was inconsistent with someone’s wants in the group”). Measuring a variable primarily through its outcomes or effects results in an indirect measurement approach, potentially overlooking the underlying mechanisms or processes responsible for generating those outcomes. Additionally, negative humor is often defined as aggressive and disparagement humor associated with negative organizational outcomes (Kong et al., 2019). However, if disparagement humor aligns with an employee’s motives, we can hypothesize that it will lead to positive outcomes. According to play-mirth theory, it is not the aggressiveness of humor that matters, but its consistency with the employee’s motives. Hence, future research should explore the roles of both positive and negative humor in relation to the concept of motive-consistency in employee satisfaction.

Play-mirth theory suggests that humor is a specific form of play and outlines the main contrasts between humor and other forms of play, but we did not examine this hypothesis experimentally. The view of humor as play can explain the close relationship between humor and laughter (Morreall, 2009). Future research endeavors could explore empirically the differences between humor and other types of play.

As with any study, the current study has several limitations. The two experiments used student convenience samples in Greece. Future studies could test the validity of our findings in a more representative sample and other countries. We do not expect cross-country or inter-age differences in the mechanisms of humor creation and appreciation; however, individuals from different cultures and age groups may vary in what they perceive as serious or playful. Future research can also utilize survey methodology to address some limitations of experiments, such as demand characteristics, sampling bias, artificiality of the environment, limited generalizability, and low external validity, though surveys have their own constraints. In addition, this approach will allow for the use of advanced statistical methods (e.g., regression analysis, structural equation modeling) instead of T-tests, providing more robust results and shedding light on subtle differences among key variables.

It is also worth noting that the present study compared predictions of the play-mirth theory with predictions of incongruity and benign violation theories, while differentiated mirth from joy, relief, sorrow, and distress. Future research could use other methodologies, measures, appraisals, and emotions to examine the play-mirth theory’s primary hypothesis in greater detail. For instance, a moment-to-moment analysis of humor creation and appreciation can confirm the role of cognitive appraisals and speed of playful turn in influencing humor intensity, using tools such as facial recognition software, EEG, eye-tracking, NVivo, or ELAN. Given the critical role of unexpectedness in both humor intensity and the creation of surprise (Roseman, 1991), it would be also interesting to explore how playful turns and motive-consistency can differentiate between mirth and surprise. Additionally, other cognitive appraisals, alongside playful turns and motive-consistency, can be tested for their potential to create or enhance humor, such as external causation (Scherer and Ceschi, 1997), and agency attributed to others (Tong, 2015).

## Data Availability

The raw data supporting the conclusions of this article will be made available by the author, without undue reservation.

## Footnotes

^1^For a more in-depth discussion of the three classic and two contemporary humor theories, please refer to section 2.1 of this paper, as well as Martin and Ford (2018) and Morreall (2020).

^2^Null Hypothesis (H1₀): Mirth is not elicited when both of the following conditions are satisfied simultaneously: (a) when an individual perceives a playful turn, and (b) the turn is considered consistent with the individual’s motive(s).

^3^Null Hypothesis (H2₀): Unexpectedness does not have a positive effect on mirth.

^4^Null Hypothesis (H3₀): Relevance does not have a positive effect on mirth.
