# Supplementary material for: Play-mirth theory: a cognitive appraisal theory of humor
Source: Front Psychol. 2024 Dec 6;15:1473742. doi: 10.3389/fpsyg.2024.1473742 (PMC11659645; doi:10.3389/fpsyg.2024.1473742)
Supplement: Supplementary file 1 [file Data_Sheet_1.docx]

Supplementary Material

# Appendix

|  | Playful Turn | Motive-Consistency | Unexpectedness | Relevance | Benign Violation | Mirth | Laugther | Joy | Relief | Negative Emotions |
| --- | --- | --- | --- | --- | --- | --- | --- | --- | --- | --- |
| Playful Turn |  |  |  |  |  |  |  |  |  |  |
| Motive-Consistency | ,774^**^ |  |  |  |  |  |  |  |  |  |
| Unexpectedness | ,446^**^ | ,478^**^ |  |  |  |  |  |  |  |  |
| Relevance | ,119 | ,171^*^ | ,051 |  |  |  |  |  |  |  |
| Benign Violation | ,467^**^ | ,509^**^ | ,183^**^ | ,276^**^ |  |  |  |  |  |  |
| Mirth | ,729^**^ | ,857^**^ | ,496^**^ | ,200^**^ | ,548^**^ |  |  |  |  |  |
| Laugther | ,729^**^ | ,865^**^ | ,508^**^ | ,160^*^ | ,535^**^ | ,871^**^ |  |  |  |  |
| Joy | ,720^**^ | ,888^**^ | ,504^**^ | ,126 | ,478^**^ | ,871^**^ | ,897^**^ |  |  |  |
| Relief | ,659^**^ | ,785^**^ | ,386^**^ | ,125 | ,527^**^ | ,753^**^ | ,787^**^ | ,812^**^ |  |  |
| Negative Emotions | -,658^**^ | -,779^**^ | -,428^**^ | -,081 | -,412^**^ | -,744^**^ | -,746^**^ | -,781^**^ | -,681^**^ |  |
| **. Correlation is significant at the 0.01 level (2-tailed). | | | | | | | | | | |
| *. Correlation is significant at the 0.05 level (2-tailed). | | | | | | | | | | |

**Appendix 1.** Correlation Matrix (Spearman's rho)

|  | Humor Manipulation | Joy Manipulation | Relief Manipulation | Sorrow Manipulation | Distress Manipulation | Mirth Intensity | Joy Intensity | Relief Intensity | Sorrow Intensity | Distress Intensity |
| --- | --- | --- | --- | --- | --- | --- | --- | --- | --- | --- |
| Humor Manipulation |  |  |  |  |  |  |  |  |  |  |
| Joy Manipulation | -,250^**^ |  |  |  |  |  |  |  |  |  |
| Relief Manipulation | -,250^**^ | -,250^**^ |  |  |  |  |  |  |  |  |
| Sorrow Manipulation | -,250^**^ | -,250^**^ | -,250^**^ |  |  |  |  |  |  |  |
| Distress Manipulation | -,250^**^ | -,250^**^ | -,250^**^ | -,250^**^ |  |  |  |  |  |  |
| Mirth Intensity | **,495^**^** | -,163^**^ | -,112^**^ | -,144^**^ | -,076^*^ |  |  |  |  |  |
| Joy Intensity | ,159^**^ | **,464^**^** | ,386^**^ | -,495^**^ | -,513^**^ | ,047 |  |  |  |  |
| Relief Intensity | ,161^**^ | ,293^**^ | **,516^**^** | -,476^**^ | -,493^**^ | ,037 | ,852^**^ |  |  |  |
| Sorrow Intensity | -,236^**^ | -,440^**^ | -,365^**^ | **,527^**^** | ,514^**^ | -,111^**^ | -,823^**^ | -,793^**^ |  |  |
| Distress Intensity | -,185^**^ | -,447^**^ | -,358^**^ | ,473^**^ | **,517^**^** | -,025 | -,782^**^ | -,752^**^ | ,875^**^ |  |
| **. Correlation is significant at the 0.01 level (2-tailed). | | | | | | | | | | |
| *. Correlation is significant at the 0.05 level (2-tailed). | | | | | | | | | | |

**Appendix 2.** Correlation Matrix (Spearman's rho)
